# Detection of Neoplastic Gastric Lesions Using Capsule Endoscopy: Pilot Study

**DOI:** 10.1155/2013/730261

**Published:** 2013-05-22

**Authors:** Byoung Yeon Jun, Chul-Hyun Lim, Wook Hyun Lee, Jin Su Kim, Jae Myung Park, In Seok Lee, Sang Woo Kim, Myung-Gyu Choi

**Affiliations:** Division of Gastroenterology, Department of Internal Medicine, College of Medicine, The Catholic University of Korea, 505 Banpo-dong, Seocho-gu, Seoul 137-701, Republic of Korea

## Abstract

*Objectives*. Capsule endoscopy is relatively noninvasive method and its use extends from the small bowel to the esophagus and colon. The aim of this study was to evaluate the feasibility and acceptability of capsule endoscopy for neoplastic gastric lesions. *Methods*. Capsule endoscopy (Pillcam ESO) was performed within 48 hours of esophagogastroduodenoscopy for eight patients who were diagnosed with gastric cancers, the size of which were less than 4 cm and who presented written consent. Patients changed position in a specified designed sequence every 30 seconds after capsule ingestion. Position change was repeated with ingestion of an effervescent agent. The rate of detection of intragastric lesions, observation of normal gastric anatomy and patient satisfaction between capsule endoscopy and esophagogastroduodenoscopy were compared. *Results*. Capsule endoscopy found four out of eight gastric lesions. The gastroesophageal junction was observed in seven of the eight cases, pyloric ring in five of the eight cases, and gastric angle in four of the eight cases. The patient satisfaction assessment questionnaire rated capsule endoscopy significantly higher than upper endoscopy in all categories. *Conclusions*. Capsule endoscopy was less effective than esophagogastroduodenoscopy and showed limited value in this feasibility study.

## 1. Introduction

Capsule endoscopy (CE) is a relatively noninvasive method that can be used to inspect the digestive tract without causing pain to the patient. CE was originally developed to inspect the small bowel, but its use has been extended to the esophagus and colon [1]. Esophageal CE has been evaluated for the diagnosis of gastroesophageal reflux disease, Barrett's esophagus, and esophageal varices [2]. As a screening tool for colon polyps and adenocarcinoma, colon CE has been compared to optical colonoscopy, and new generation colon CE has recently been evaluated in a large multicenter trial [3–5]. However, there are not many CE studies of stomach lesions.

Standard esophagogastroduodenoscopy (EGD) is a most effective diagnostic method for upper gastrointestinal lesions including esophageal disease. However, patients often complain of discomfort during inspection and are reluctant to have repeated endoscopy for follow-ups. Transnasal endoscopy seems to be more compliable than standard EGD [6, 7] but is not used widely. Therefore, CE could be considered as a diagnostic tool if we can determine its diagnostic accuracy.

Because of the high incidence of gastric cancer, endoscopy for gastric cancer is frequently performed in Republic of Korea, even in patients without symptoms. In general, asymptomatic individuals tend to prefer simpler and less invasive procedures, by which a higher screening rate could be achieved. Therefore, the advent of capsule endoscopy for the stomach is expected. The aim of this study was to evaluate the feasibility and acceptability of capsule endoscopy for gastric lesions.

## 2. Materials and Methods

This study was designed as a prospective, single-center open-label trial and was approved by our Institutional Review Board (clinical trial no. KCMC08ER99). Enrollment of patients in the study was based on the following inclusion criteria: (1) patient age between 18 years and 65 years, (2) patients that were diagnosed with gastric cancer, less than 4 cm, during EGD at Seoul St. Mary's Hospital from October 2008 to December 2009, and (3) patients who presented written consent. The exclusion criteria for participation in this study were (1) dysphagia, (2) esophageal diverticula, (3) esophageal stricture or upper gastrointestinal tract stricture, (4) a history of surgery except appendectomy or cholecystectomy, (5) gastrointestinal bleeding, (6) mechanical bowel obstruction, (7) bowel perforation, (8) pregnancy, (9) implanted electric medical device such as an artificial heart pump, (10) scheduled MRI within 7 days of CE, (11) psychological disease, and (12) alcohol or drug dependence.

CE with PillCam ESO (Given Imaging, Yokneam, Israel) was performed within 48 hours of EGD. The PillCam ESO is a disposable video capsule and 26 × 11 mm in size. It is equipped with two metal oxide silicon chip cameras placed at both ends, and each camera is surrounded by light-emitting diodes. It can acquire up to seven images per second per camera for 20 minutes and is powered by two silver oxide batteries. Images are transmitted to skin sensors by an ultrahigh frequency radio telemetry system and stored on a hard disk. Images generated have a 140° viewing field, and the depth of visibility is up to 30 mm.

The examinees were injected with 5 mg of cimetropium bromide intramuscularly [8] and then swallowed 10 mL of simethicone solution (20 mg/mL) [9, 10] and the capsule endoscope with 100 mL of water in an upright position. Two minutes after swallowing, they changed positions every 30 seconds in sequence from the supine position to left lateral, prone, left lateral, supine, right lateral, supine, right lateral-head up tilt, and right lateral-head down tilt positions. The examination was repeated with ingestion of 4 g of sodium bicarbonate as an effervescent agent. An examiner who was blinded to the results of the EGD observed the images in real-time and documented the findings. CE images were reviewed using Rapid 5 Access software (Given Imaging, Yokneam, Israel).

The rate of detection of intragastric lesions by CE was evaluated as the primary end point. As secondary end points, observation of the normal gastric anatomy (gastroesophageal junction, gastric angle, and pylorus) and satisfaction of patients were evaluated and compared with EGD. The examinees were questioned about their satisfaction with CE and EGD after completion of both examinations. Difficulty in swallowing, pain during examination, discomfort during examination, pain after examination, and discomfort after examination were scored from 1 to 5 (1, none; 2, negligible; 3, slight; 4, severe; 5, difficult to endure). General ease of examination was scored from 1 to 4 (1, very comfortable; 2, comfortable; 3, uncomfortable; 4, very uncomfortable). The time of daily activity influenced after examination was scored from 1 to 5 (1, 0–2 hours; 2, 3-4 hours; 3, 5-6 hours; 4, 7-8 hours; 5, more than 8 hours).

Patient satisfaction is presented as a mean ± standard deviation. A Wilcoxon signed-rank test was used to compare the difference in patient satisfaction between the two procedures. *P* < 0.05 was considered as statistically significant. Statistical analysis was performed using SAS for Windows software (version 8.02, SAS Institute, Cary, NC, USA).

## 3. Results

**Figure 1:**
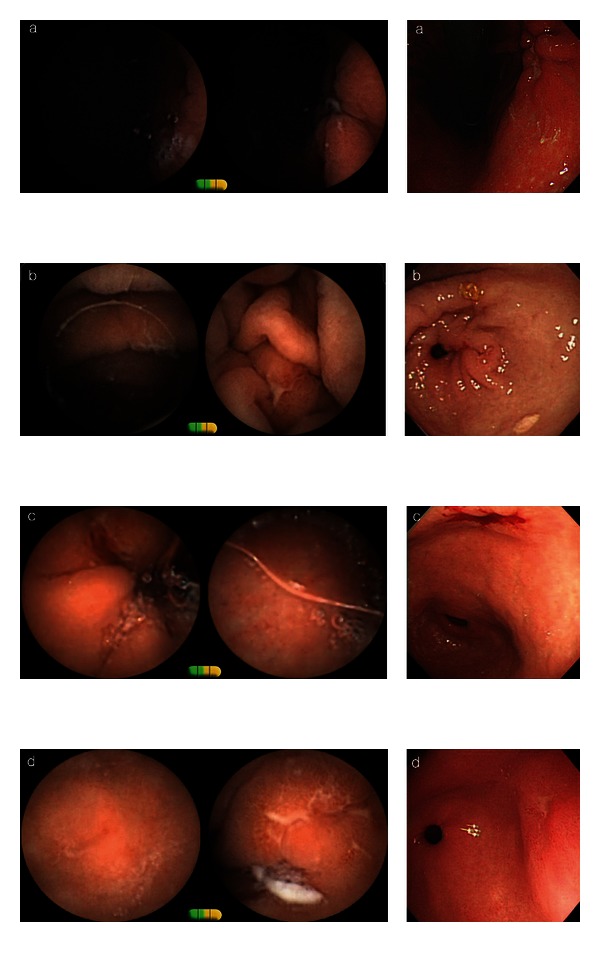
Findings of EGD (right) and CE (left) of patient (a–d).

**Figure 2:**
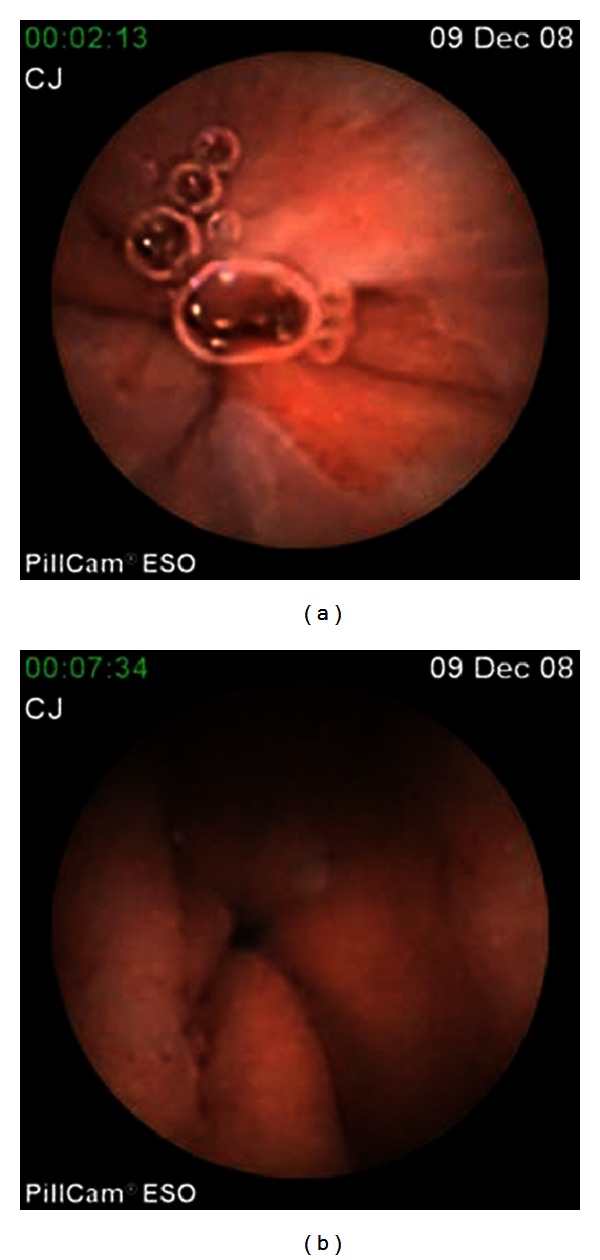
Examples of normal structures of stomach by CE: gastroesophageal junction (a) and pylorus (b).

The male-to-female patient ratio was 5 : 3. Median age was 61 (31–76) years. The endoscopic lesions found by EGD were one case of early gastric cancer (EGCa) type I, five cases of EGCa type IIc, one case of EGCa type III, and one case of advanced gastric cancer Borrmann type III. Median size of lesions was 25 (15–40) mm. CE found four of the eight lesions (Figure 1). The locations of lesions were in the lower body in one case and the antrum in three cases. The gastroesophageal junction (GEJ) was observed in seven of the eight cases. The pyloric ring was observed in five of the eight cases (Figure 2). The gastric angle was observed in four of the eight cases (Table 1).

The patient satisfaction assessment with the CE and EGD is shown in Table 2. The patient satisfaction assessment questionnaire rated the CE significantly higher than the upper endoscopy in all categories, including pain and discomfort during and after the procedure, overall convenience, and missed time from work. No adverse events, including capsule retention, were reported with either procedure in this study during the 2-week follow-up period.

## 4. Discussion

CE was introduced in 2000, and since then, indications for small bowel diagnostics have been established [11]. However, by contrast, application of CE to the stomach has been considered as difficult, because spontaneous capsule passage would not provide sufficient information in the large space of the gastric cavity. In this study, we assessed the feasibility of CE for the diagnosis of gastric lesions.

In this study, the diagnostic rate of CE for gastric lesions was 50%. This rate is not sufficient as a screening examination. In a recent Japanese study, the sensitivity for localized lesions (erosion, cancer, and polyps) was very low, so the investigators concluded that capsule endoscopy was not sufficient to diagnose gastric disease [12].

When the CE data were reviewed after we unblinded the EGD results, we found two more lesions. One of additionally detected lesions was a type I EGCa seen at the greater curvature of the high body. The other additional lesion was a type IIa EGCa seen at the posterior wall of the antrum. The first missed lesion was covered with bubbles so that it was barely seen but could be identified. Effervescent agents were used to distend the stomach according to procedures such as those used in the acquisition of an upper gastrointestinal series of images (barium swallow) and 3D stomach CT. As a result, the visual field can be extended, and the spaces between folds can be visualized. However, the bubbles generated can camouflage the lesions. In this respect, a surfactant such as simethicone can improve visibility by reducing air bubbles in the stomach. To decrease the number of bubbles, we could attempt to increase the quantity of simethicone or administer it again after using the effervescent agent. In a recent meta-analysis, investigators concluded that supplemental use of simethicone before endoscopy decreases the number air bubbles, although there was insufficient data to determine whether this was statistically significant [10]. The second missed lesion was seen clearly when reviewed, so that it was considered that the examiner simply missed it.

Although the stomach was expanded with water and an effervescent agent, expansion was not as sufficient as when we observed the lesion with endoscopic insufflation. It is likely that the lesion evaded detection by being hidden between the folds. More experience may enable investigators familiar with the CE views to improve diagnostic yield.

We attempted to identify landmarks in the stomach, which included the GEJ, gastric angle, and pylorus. The GEJ and pylorus are recognizable by shape; so we could determine whether they were observed or not. However, the anatomy of the gastric angle is obscure, perhaps because of an incomplete expansion of the stomach and shallow depth of view. Structures that we can identify easily, such as the GEJ and pylorus, can provide information regarding the whereabouts of the CE. Based on sequence of the patients' positions and estimated position of the CE, we could verify the visualization of the fundus with a relatively high probability. However, insufficient insufflation and shallow penetration by the light source prevented overall visualization, such that it was not possible to determine if the fundus was visualized with clinical significance. This uncertainty might be reduced if changing the patients' position can be directed by real-time CE.

In general, the stomach is not sufficiently expanded even with gas-foaming agent, and CE remains at the dependent surface with one end trapped into the mucosa of the body and the visible depth not sufficiently deep. The cardia and fundus were barely recognized at any time. Therefore, except when we saw the pylorus when the CE could be located near the antrum, the exact location of the CE was uncertain most of the time, and the subjective percentage of the visible mucosa could not be calculated. The lesions found were at the lower body in one case and at the antrum in three cases. These findings suggest that capsules reside on the greater curvature side of the body or the antrum most of the time.

To resolve the drawbacks of CE, especially in a large space like the stomach, a new tool for maneuvering a CE in the human digestive tract has been developed. The wireless capsule was manipulated with magnetic paddles and observed simultaneously with a video gastroscope in one subject. Spinning, rotation, and somersaults were successfully achieved. Movements up and down the axis of the stomach were successful. The position of the capsule in the stomach with the orientation and movement of an external handheld magnet was assessed by direct observation of the gastroscope [13]. Self-propelling CE using a magnetic field was also attempted in an animal model. The vibration generated in a magnetic field was transmitted to a fin, and it allowed the vibration of the magnet to be converted into a propelling force. One liter of water was administered to a sedated dog by using EGD. The capsule was inserted through an overtube under EGD observation, and clear images could be obtained [14]. In a feasibility study of the magnetically maneuverable capsule in healthy volunteers, investigation showed that both the cardia and the pylorus were inspected, and 75% or more of the gastric mucosa was visualized (in seven out of ten subjects). Even though they used a magnetically maneuverable capsule with an external magnetic paddle, they concluded that visualization of the mucosa was incomplete, because of fluid blocking the view of the most apical parts of the fundus and partially because of suboptimal gastric distension [15]. In another feasibility study using low-level magnetic fields produced by the magnet of a guidance system that resembles conventional MRI, the subjects drank 1300 mL of water within 1 hour of the examination. The technical success rate was 98%. Examiners assessed that the antrum, body, fundus, and cardia were fully visualized in 98%, 96%, 73%, and 75%, respectively. Mean duration of examinations was 30 (range 8–50) minutes [16].

Considering the results of the studies described above, distension of stomach with a large amount of water is thought to provide more volume than less water combined with effervescent agents. To address the aspect of visual depth, a stronger source of light could afford more coverage of gastric mucosa. If effervescent agents are to be used, surfactant such as simethicone after effervescent agents may help to reduce air bubbles attached to mucosal surface.

## 5. Conclusion

Diagnostic yield was not satisfactory when we inspected the stomach with CE. However, by combining effective methods of stomach distension and maneuvering, the diagnostic rate provided by CE may be improved and allow a relatively comfortable screening modality for gastric cancer.

## Figures and Tables

**Table 1:** Type and location of stomach lesions and the result of capsule endoscopy.

No. of patients	Sex	Age	Type of lesion by EGD	Location	Size (mm)	Lesion found by CE	GEJ	Angle	Pyloric ring
1	F	60	EGCa IIc	Lower body PW	30	o	x	o	x
2	M	76	EGCa I	High Body GC	40	x	o	x	o
3	M	61	EGCa III	Prepyloric antrum PW	20	o	o	o	o
4	F	61	EGCa IIc	Antrum PW	40	x	o	x	x
5	F	31	EGCa IIc	Lower body AW	30	x	o	o	o
6	M	62	EGCa IIc	Antrum PW	15	o	o	x	o
7	M	70	Borrmann III	Lower body PW	20	x	o	x	x
8	M	48	EGCa IIc	Antrum PW	15	o	o	o	o

**Table 2:** Result of patients' satisfaction assessment with PillCam ESO capsule endoscopy (CE) and upper endoscopy (EGD) measured using a 1 to 5 discrete scale, with 5 being the most comfortable/easy and 1 being the least comfortable/easy score^a^.

	Difficulty of swallowing	Pain during examination	Discomfort during examination	Pain after examination	Discomfort after examination	Overall convenience of examination	Time influenced by examination
EGD (Mean ± SD)	3.75 ± 1.28	2.38 ± 1.51	3.13 ± 1.64	2.75 ± 1.51	2.38 ± 1.64	3.00 ± 0.76	2.38 ± 1.69
CE (Mean ± SD)	1.88 ± 0.84	1.00 ± 0.00	1.25 ± 0.71	1.00 ± 0.00	1.00 ± 0.00	1.75 ± 0.46	1.00 ± 0.00
Mean difference (EGD−CE)	1.87	1.38	1.88	1.75	1.38	1.25	1.38
*P* value (2-tailed)	0.025	0.042	0.041	0.026	0.041	0.026	0.038

^a^Wilcoxon signed-rank test.
